# A Meta-Analysis of the Effects of Teacher Personality on Teacher Effectiveness and Burnout

**DOI:** 10.1007/s10648-018-9458-2

**Published:** 2019-01-02

**Authors:** Lisa E. Kim, Verena Jörg, Robert M. Klassen

**Affiliations:** 1 Department of Education, University of York, York, UK; 2 Johannes Gutenberg University Mainz, Mainz, Germany

**Keywords:** Teacher personality, Job performance, Teacher effectiveness, Burnout, Big Five

## Abstract

The question of what makes a good teacher has been asked by practitioners, policymakers, and researchers for decades. However, there is no guiding framework about which qualities are important for teachers. Thus, it is necessary to examine these qualities using a recognized framework and to summarize the previous literature on this topic. We conducted a meta-analysis on the 25 studies (total *N* = 6294) reporting the relationships between teacher Big Five personality domains (openness, conscientiousness, extraversion, agreeableness, and emotional stability) and two teacher job-related outcomes (i.e., teacher effectiveness and burnout). Furthermore, the influence of three moderators was assessed, namely, the type of teacher effectiveness measure (i.e., evaluations of teaching, student performance self-efficacy, classroom observation, and academic achievement), source of personality report (i.e., self-report vs other-report), and the instructed educational level (i.e., elementary, secondary, and tertiary). Overall, teacher Big Five domains (except for agreeableness) were positively associated with teacher effectiveness, especially for evaluations of teaching. Furthermore, teacher emotional stability, extraversion, and conscientiousness were negatively associated with burnout. Other-reports of teacher personality were more strongly associated with outcomes than self-reports. There were no differences in the strength of the associations between the educational levels. The need for using common descriptors in teacher research as well as practical implications of the findings for teacher personality measurement is discussed.

Teachers are prominent figures in the educational system both statistically and in their potential for influencing educational outcomes. According to the World Bank EdStats (2017), there are 84.23 million teachers in the world across educational levels: pre-primary (9.36 million), elementary (30.27 million), secondary (32.12 million), and tertiary (12.49 million). Furthermore, an additional 68.8 million teachers will need to be recruited by 2030 to provide every child with elementary and secondary education (UNESCO Institute for Statistics 2016). Teachers are important drivers of student success in the immediate term, such as academic success (Hattie 2009), as well as in the future, such as college attendance and labor market earnings (Chetty et al. 2014). Additionally, it is important to retain teachers given that there is a shortage of teachers in many countries, such as the USA (Sutcher et al. 2016), Australia (Buchanan et al., 2013), and the UK (White et al. 2006). However, two questions still remain among practitioners, policymakers, and researchers: *what are the personal characteristics of effective teachers* and *what are the personal characteristics of teachers with low burnout tendencies?* More specifically, *what are the relationships between teacher personality and the job-related outcomes of teacher effectiveness and burnout?* No previous study has examined the meta-analytic association between teacher personality using a Big Five framework and teacher effectiveness and burnout. In this light, the current study aims to examine the extent to which each of the Big Five personality domains is associated with measures of teacher effectiveness and burnout.

## Personality Model and Assessment

Personality describes the unique psychological qualities that influence individuals’ behaviors, thoughts, and feelings across situations and times (Roberts and DelVecchio 2000; Roberts and Jackson 2008). To understand the construct of personality, researchers have proposed numerous personality frameworks which have varying levels of evidence of reliability and validity. Examples of personality frameworks include the HEXACO (Ashton and Lee 2007), Myers-Briggs Type Indicator (Myers et al. 1998), and 16 personality factors (Cattell et al. 1970). However, the Big Five is the dominant personality framework (John et al. 1991; John et al. 2008), which is underpinned by the lexical hypothesis. According to this hypothesis, socially relevant and salient descriptions that are used frequently to describe individuals and distinguish one from another are retained in our natural language (see Saucier and Goldberg 1996 for a review). Numerous researchers (e.g., Allport and Odbert 1936; Cattell 1943) studied these descriptors as the basis to creating a scientific taxonomy of personality traits, which led to replicated findings that there are five domains underlying one’s personality: openness (creative, curious, cultured), conscientiousness (organized, responsible, reliable), extraversion (sociable, assertive, energetic), agreeableness (kind, cooperative, trustful), and emotional stability (calm, secure, unemotional). Multiple scales are available to measure the Big Five domains, such as the Big Five Inventory (BFI; John et al. 1991; John et al. 2008), Ten-Item Personality Inventory (TIPI; Gosling et al. 2003), and the Mini-Markers (Saucier 1994).

The five-factor model (FFM) is a similar model to the Big Five framework. Unlike the Big Five framework, however, the FFM is derived from empirical analyses of questionnaires. Costa Jr and McCrae (1976) cluster-analyzed the 16 personality factors (16PF; Cattell et al. 1970) to initially create three domains: neuroticism, extraversion, and openness. Agreeableness and conscientiousness were later added to create five factors that were similar to the Big Five framework domains (McCrae and Costa Jr. 1987). The two models are very similar (DeYoung et al. 2007), though there are slight differences such as the FFM’s broader definition of openness by including elements of unconventionality and behavioral flexibility (Costa and McCrae 1978). Multiple scales are available to measure the FFM domains, such as the 240-item Revised NEO Personality Inventory (NEO-PI-R; Costa and McCrae 1992), 60-item NEO Five Factory Inventory (NEO-FFI; Costa and McCrae 1992), and 12-item NEO Personality Inventory (NEO-PI; Costa and McCrae 1985). Given the high similarity between the two models, meta-analyses often combine the domains from the two frameworks (e.g., Oshio et al. 2018; Parks-Leduc et al. 2015; Richardson et al. 2012; Vedel 2014).

Personality measures, including those based on the Big Five framework and the FFM, vary in multiple ways, such as accessibility, length, and the specificity of the domains. For example, TIPI is a publicly available 10-item Likert-scale-based measure of the Big Five framework (measuring five domains), whereby participants rate the degree to which they agree a pair of adjectives accurately describes them. On the other hand, NEO-PI-R is a commercial 240-item Likert-scale-based measure of the FFM (measuring five domains and each of the domain’s six facets), whereby participants rate the degree to which they agree each statement accurately describes them. The choice to use one measure over another can depend on factors such as test time restrictions and the desired specificity measurement of the personality domain or facet. The Big Five framework and its various measures are used in numerous fields, such as in health psychology, industrial-organizational psychology, and clinical psychology (see Ozer and Benet-Martínez 2006 for a review).

In education, personality researchers have primarily focused on understanding student personality. Multiple meta-analyses have reported that student Big Five domains are positively associated with academic achievement, predicted most strongly by conscientiousness, with effect sizes ranging from .22 to .27 (O’Connor and Paunonen 2007; Poropat 2009; Richardson et al. 2012; Trapmann et al. 2007). The Big Five framework has also been useful in predicting other educational outcomes, such as academic motivation (Komarraju et al. 2009), academic dishonesty (Giluk and Postlethwaite 2015), and career decision-making (Martincin and Stead 2015).

On the other hand, despite a long history of interest in the personality profile of effective teachers (Dodge 1943), there has been a lack of studies on teacher personality, especially using established personality theories. In Göncz’s (2017) review of teacher personality research, he outlined five categories of studies that have been conducted in this area: (a) descriptions of teacher types, (b) qualities of “desirable” as opposed to “undesirable” teachers, (c) impact of teacher professional behavior (e.g., teaching methods), (d) impact of teachers’ professional identity (e.g., self-concept), and (e) teacher personality within a personality framework. Many of the studies of the second category are qualitative. For example, Witty (1947) examined 12,000 letters from primary and secondary school students describing “the teacher who has helped me most.” A tabulation of the repeated characteristics or traits showed that the most mentioned traits included “cooperative, demographic attitude,” “kindliness and consideration for the individual,” and “patience.” Other studies have evaluated teacher’s personality on a global scale, such as by asking “How do you feel about the instructor as a person” on a Likert scale from “Doesn’t appeal to me at all” to “Terrific; a great person” (Jones 1989). However, these lists and questions lack robust theoretical and empirical background. We agree with Göncz, who argued that more studies using established personality frameworks should be conducted, given it is the most promising avenue to building comprehensive teacher personality theories.

## Job-Related Outcomes: Teacher Effectiveness and Burnout

One must identify the criteria against which to evaluate which are the important teacher personal characteristics; that is, to establish its criterion validity (Gordon et al. 2006). Among many job-related outcomes integral to the teaching profession, teacher effectiveness and burnout capture important cross-sections of teachers’ professional impact and experience. As such, this meta-analysis will focus on how teacher personality is associated with these two criterion outcomes.

### Teacher Effectiveness

Although various conceptualizations of teacher effectiveness have been proposed throughout the years (e.g., Barr 1939; Cheng and Tsui 1999; Doyle 1977; Muijs 2006), we still do not have a consensual definition (see Goe et al. 2008 for a review). Yet, teacher effectiveness has been the focus of educational impact research, including the $45 million Measures of Effective Teaching (MET) project. The MET study was designed to assess and promote effective teaching, and the study included three measures of teacher effectiveness to achieve this aim: student evaluations of teaching, classroom observation, and student academic achievement (Kane et al. 2014). What is agreed, however, is that these are the most common teacher effectiveness measures (see Goe et al. 2008 for a review of teacher effectiveness measures).

Student evaluations of teaching aim to capture students’ perception of the classroom and the teacher. Of many measures of student evaluations, the Tripod perception survey (Ferguson 2010) is the most popularly used tool in the USA (Wallace et al. 2016), which consists of seven dimensions, including care (level of interpersonal relationship support), confer (level of soliciting and inviting students’ perspectives), and captivate (level of interest and relevancy of teaching). Tripod is adapted for two educational levels—elementary and secondary. In higher education, one of the most widely used student evaluation tools is the Student Evaluations of Educational Quality questionnaire (SEEQ; Marsh, 1984), which contains seven domains assessing the level of university teaching, including group interaction (stimulation of classroom discussions and enquiry), enthusiasm (level of enthusiasm in the classroom), and organization (clarity of instructions, preparation of course material). Some researchers argue the validity of student evaluations as a marker of student learning (see Clayson 2009 for a review). However, its frequent use by practitioners and policymakers—including to provide feedback to instructors (Taylor and Tyler 2012) and make high-stake decisions (Glazerman et al. 2010; Murray et al. 1990)—compels researchers to consider it as a teacher effectiveness measure.

Classroom observations are one of the most labor-intensive measure of teacher effectiveness. It often requires training observers to code an individual’s effectiveness in the classroom over multiple lessons. The most common classroom observation tools are Classroom Assessment Scoring System (CLASS; Pianta et al. 2008) and Danielson’s Framework for Teaching (FfT; Danielson 2013), both of which were implemented in the MET study. CLASS offers an observation tool adapted for different educational levels (i.e., infant, toddler, lower elementary, upper elementary, and secondary), whereas Fft offers an observation adapted for different subject areas (i.e., mathematic and English Language Arts) as well as a generic subject area.

Student academic achievement, a marker for the level and progress of student learning, is arguably the most common measure of teacher effectiveness and often considered as a gold standard outcome to evaluate the influences of various factors. The type of achievement outcome used can vary, including standardized test scores, such as the Texas Assessment of Knowledge Skills test (e.g., Garcia et al. 2011), as well as non-standardized scores, such as school GPA or ACT scores (e.g., Radmacher and Martin 2001), and university subject marks (e.g., Kim and MacCann 2018).

Student performance self-efficacy (PSE, also known as academic performance self-efficacy) is also a teacher effectiveness measure, which is students’ perception of their capability to perform academically (Shell & Husman, 2001). With a strong theoretical background, particularly that from Bandura (1977) within social cognitive theory (Bandura, 1986), PSE is mostly used in student non-cognitive research to understand its effect on student academic functioning (e.g., Bandura et al. 1996) and how it can be enhanced (e.g., Schunk & Ertmer, 2000). PSE is most often measured by asking students to report a mark that they expect to receive in a particular subject (e.g., Shell & Husman, 2001; Kim et al., 2018; Kim and MacCann 2018) or their confidence that they will perform well academically on specific tasks, such as note taking, test taking, and writing papers (e.g., Chemers et al. 2001). Each of the above-mentioned teacher effectiveness measures can contribute uniquely to providing a holistic picture of teacher effectiveness (Muijs 2006; Rockoff & Speroni, 2010).

### Burnout

Teaching can be stressful (Kyriacou 2001) and can result in burnout, which is considered a consequence of unsuccessful execution of coping strategies resulting in prolonged stress (Guglielmi and Tatrow 1998; Vandenberghe and Huberman 1999). Burnout is a syndrome of emotional exhaustion (emotional overextension and exhaustion), depersonalization (uncaring attitude towards others), and reduced personal accomplishment (low levels self-competence and satisfaction with own work; Maslach et al. 2001). The most commonly used measure of burnout is the Maslach Burnout Inventory (MBI; Maslach et al. 1996), which measures the frequency of one’s experiences of burnout symptoms from *never* to *every day*. The MBI was originally developed for the human service occupations (MBI-Human Services Survey) but now also has the MBI-Educators Survey (MBI-ES) and the MBI-General Survey (MBI-GS; Maslach et al. 2001). The MBIs have validity evidence for use in both normal and clinical populations (Schaufeli et al. 2001) as well as in different occupational groups and cultures (Schutte et al. 2000). Within the education field, the MBI-GS and MBI-ES have been used across elementary, intermediate, secondary, and university educators (e.g., Byrne 1991; Byrne 1993; Kokkinos 2006) as well as teachers from across cultures (e.g., Schwarzer et al. 2000; Van Horn et al. 2004).

Burning out is a particular risk for individuals working with other people in some capacity (Maslach et al. 2001). The teaching profession is composed of long episodes of engagement and interactions with students and staff and high workload, which can present opportunities for emotional draining and discouragement (Chang 2009). The significance of the problem is highlighted by a meta-analytic finding that burnout is associated with absenteeism, turnover, and job performance (Swider and Zimmerman 2010).

## Job-Related Outcomes: Teacher Personality

Previous studies have examined the associations between teacher factors (including teacher personality) and teacher effectiveness and teacher burnout to various degrees. Some of the most notable teacher effectiveness findings are those from Hattie, who updated his original meta-analysis of meta-analyses (Hattie 2009) to list over 250 factors that impact on student academic achievement (see Visible Learning Plus 2018 for a full list of factors and effect sizes). These factors are categorized into seven sources of impact: (a) teaching (teaching/instruction strategies, student learning strategies, and implementation methods), (b) teacher, (c) student, (d) school, (e) home, (f) curricula, and (g) classroom. The teacher source is further divided into teacher attributes, teacher-student interactions, and teacher education. The strengths of the effect sizes vary among the 14 identified teacher factors, ranging from teacher performance pay (*d* = .05) to teacher estimates of student achievement (*d* = 1.29). Teacher personality is also included in the list of factors (*d* = 0.24), which is greater than teacher verbal ability (*d* = 0.22). We cannot comment on the specifics of the study as details, such as the search terms and the studies that were included in the meta-analysis, are not available. Klassen and Tze (2014) also conducted a meta-analysis between teacher personality and teacher effectiveness and found a small but significant relationship (*r* = .08, *p* < .05). However, like Hattie, they also considered teacher personality as a unidimensional construct, which is not in line with dominant personality theories, including the Big Five framework.

In Chang’s (2009) review of the teacher burnout literature, she identifies that there are generally three sources contributing to burnout: individual factors (e.g., gender, years of experience, personality, coping strategies); organizational factors (e.g., work demands, school socioeconomic status/culture, organizational rigidity); and transactional factors, which are interactions between individual and organizational factors (e.g., teachers’ judgements of student misbehaviors; perceived support from principal, peers, and administration). Although teacher personality was one of the identified factors, only non-Big Five characteristics were outlined (e.g., low hardiness, type-A personality, lower self-esteem, and high expectations). Systematic reviews and meta-analyses have also linked teacher burnout with other factors, such as teacher emotional intelligence (Mérida-López and Extremera 2017), teacher self-efficacy (Brown 2012), student motivation (Shen et al. 2015), and student misbehavior (Aloe et al. 2014). However, there is yet a meta-analysis on the possible link between teacher personality and burnout.

### Teacher Effectiveness

Teacher effectiveness is a measure of job performance in the teaching profession (Gordon et al. 2006), as it can capture the impact the teacher has had in performing their job. Thus, we can use both research from organizational psychology and educational psychology to hypothesize about the potential associations each of the Big Five domains (conscientiousness, emotional stability, extraversion, agreeableness, and openness) may have with teacher effectiveness.

Individuals with high levels of conscientiousness tend to be oriented towards being achievement-focused, highly responsible, and organized (John et al. 2008). As such behaviors are helpful in successful completing tasks, conscientiousness has typically been the strongest predictor of job performance in meta-analyses (Barrick and Mount 1991; Salgado 2003). Particularly, the facets of achievement striving (drive to achieve goals), dutifulness (sense of moral conscience), and self-discipline (ability to begin and complete tasks) are the facets most positively associated with job performance (Judge et al. 2013). In the teaching profession, Klassen et al. (2017) identified that organization and planning were important personal characteristics for effective teachers. This group of characteristics captured one’s ability to manage competing priorities and their time, and display general organization skills, which are conceptually very similar to elements of conscientiousness, and thus indicating that conscientiousness may also be an important predictor of teacher effectiveness. Empirical study findings also support that teacher conscientiousness is positively associated with teacher effectiveness measures (e.g., Garcia et al. 2011; Kim et al. 2018; Kim and MacCann 2018; Murray et al. 1990). For example, Patrick (2011) found that overall teacher evaluations at tertiary level were predicted most strongly by high levels of teacher conscientiousness, even after controlling for students’ previous learning and expected grade. Given that teaching requires planning and independence to complete tasks and to impart knowledge to others, we expect that teacher conscientiousness will be positively associated with teacher effectiveness (H1a).

Individuals with high levels of emotional stability are calm, secure, and tolerant of stress (John et al. 2008) and these qualities can be helpful to establish trust and credibility with others, particularly for jobs requiring interpersonal interactions (Mount et al. 1998). Emotional stability is typically the second strongest predictor of job performance in meta-analyses (Barrick and Mount 1991; Salgado 2003). Particularly, the facets of impulsiveness (low self-control), depression (guilt, sadness), and anxiety (fear, worry) are the facets most negatively associated with job performance (Judge et al. 2013). Teachers are emotional contagions; their emotions displayed in the classroom can be transmitted to students (Frenzel et al. 2018; Hatfield et al. 1994). Students too can become anxious and nervous, when observing an anxious and nervous teacher. In turn, students’ perceptions of the school and the teacher and their academic ability may be affected. Thus, we expect that emotional stability will be positively associated with teacher effectiveness (H1b).

Individuals with high levels of extraversion display their energy outwards (Fielden et al. 2015), which is positively associated with higher levels of communication, sensitivity, disclosure, and provision of social support (Wilt and Revelle 2009). Barrick and Mount (1991) found in their meta-analysis that extraversion was positively associated with job performance for occupational groups requiring interpersonal interactions (i.e., managers and sales representatives; *p̂* = 0.18, 0.15, respectively) in a way that did not extend to other occupational groups (*p̂* = − 0.05 to 0.09). Within teaching, Isaacson et al. (1963) found that university teaching fellows are rated as high in surgency (similar to extraversion) received high ratings in individual rapport and group interaction in teaching evaluations. Teaching requires assertiveness and social interaction, for which gregariousness and sociability may be an advantage. Thus, we expect teacher extraversion will be positively associated with teacher effectiveness (H1c).

Individuals with high levels of agreeableness are kind, caring, and helpful (John et al. 2008). Such qualities can be helpful to create positive and warm environments, which students require in learning (Pianta and Hamre 2009). For jobs requiring interpersonal interactions, like teaching, agreeableness is the strongest predictor of job performance (Mount et al. 1998). Agreeableness is conceptually aligned with the empathy aspect of empathy and communication from Klassen et al.’s (2017) identified effective teacher characteristics. This quality measures one’s level of actively listening and engaging in a conversation, seeking advice, and adjusting their communication style. Empirical study findings from the teaching domain also support the importance of agreeableness in students’ perceptions of effective teaching. At tertiary level, teacher agreeableness was the strongest predictor of overall teacher evaluation, individual rapport, and enthusiasm (Kim and MacCann 2018). At secondary level, teacher agreeableness was the strongest predictor of students’ report of teacher personal support (Kim et al. 2018). The quality of being warm and sensitive with students, staff, and parents is a quality required daily in teachers as they come to interact with them. Thus, we expect teacher agreeableness will be positively associated with teacher effectiveness (H1d).

Individuals with high levels of openness are intellectually curious, creative, and liberal (John et al. 2008). Openness is one of the weakest predictors of job performance, although the values (readiness to reexamine opinions and values, liberal) facet of the domain still seems to be important in predicting job performance (Judge et al. 2013). Openness is also aligned with Klassen et al.’s (2017) adaptability aspect of the resilience and adaptability domain, which measures one’s level of flexibility in lesson delivery and persistence under pressure. Patrick (2011) reported that teacher openness was the strongest predictor of overall class ratings, controlling for students’ previous learning and expected grade. Similarly, Kim and MacCann (2018) found that teacher openness was the strongest predictor of overall course evaluations. The ability to be flexible in teaching and engaging with students’ ideas and opinions is a highly valued quality in teachers. We expect that teacher openness will be positively associated with teacher effectiveness (H1e).

### Burnout

Burnout occurs particularly in jobs requiring interpersonal interactions, which teaching does (Maslach et al. 2001). The association between personality and burnout has been extensively studied in multiple professions using meta-analytic approaches (e.g., Alarcon et al. 2009; Swider and Zimmerman 2010). However, a meta-analysis on the association between personality and burnout in the teaching profession has not been conducted, although reducing teacher burnout is a prominent topic of discussion among researchers, practitioners, and policymakers (see Iancu et al. 2018 for a review on burnout interventions). Meta-analytic findings from other fields as well as teacher studies allow us to form hypotheses about how the Big Five domains (emotional stability, extraversion, conscientiousness, agreeableness, and openness) may be associated with teacher burnout, with the strongest effects from the first three domains.

Numerous meta-analyses have shown that emotional stability is the strongest correlate of burnout (Alarcon et al. 2009; Swider and Zimmerman 2010). Similarly in teaching, Kokkinos (2007) examined the associations between teacher Big Five domains and the three domains of the MBI-ES and found that high levels of teacher emotional stability were associated with low levels of emotional exhaustion, depersonalization, and high levels of reduced personal accomplishment. Individuals low in emotional stability, by definition, tend to experience negative, distressing emotions. Thus, we expect teacher emotional stability will be negatively associated with job burnout (H2a).

Meta-analyses have also shown that extraversion is the second strongest correlate of burnout, especially with the strongest effect for the reduced personal accomplishment domain (Alarcon et al. 2009; Swider and Zimmerman 2010). Individuals with low levels on this domain tend to focus on negative aspects of situations (Suls et al. 1998), encode and recall negative information (Watson and Clark 1984), and use ineffective coping strategies such as wishful thinking, withdrawal, and emotion-focused coping (Connor-Smith and Flachsbart 2007). Thus, we expect teacher extraversion will be negatively associated with teacher burnout (H2b).

Conscientiousness is also a strong correlate of burnout, and is especially strongly related with the depersonalization domain (Alarcon et al. 2009; Swider and Zimmerman 2010). Conscientiousness, by definition, describes approach-oriented individuals, thus is not compatible with behaviors associated with withdrawal. In teaching, Kokkinos (2007) found that teacher conscientiousness was particularly important for two of the three domains of burnout: depersonalization and reduced personal accomplishment. That is, teachers who are less strong willed and persistent in pursuing goals are more prone to build emotional callousness, withdraw themselves from situations, and become cynical to avoid further stress and strain. Furthermore, given the link between conscientiousness and job performance (Judge et al. 2013) and job satisfaction (Judge et al. 2002), individuals high in this domain are less likely to feel that they are not achieving in their work. Thus, we expect teacher conscientiousness will be negatively associated with burnout (H2c).

Agreeableness is a moderate correlate of burnout (Alarcon et al. 2009; Swider and Zimmerman 2010). Individuals with high levels on this domain are warm and supportive; as such, they tend to build successful interpersonal relationships at work (Organ and Lingl 1995). They tend to be cooperative, and their kind behaviors are often reciprocated by colleagues (Bowling et al. 2004). The positive work environment that they create diminishes the likelihood that they would feel isolated at work. Thus, we expect teacher agreeableness will be negatively associated with burnout (H2d).

Openness is usually the weakest correlate of burnout (Alarcon et al. 2009; Swider and Zimmerman 2010). Individuals with high levels on this domain are intellectually curious and open-minded. In effect, they view work difficulties as opportunities for personal growth rather than a hindrance (Zimmerman 2008). There are inconsistent findings on the association between openness and coping strategies; some studies report significant associations with problem-focused coping strategies (e.g., Strutton et al. 1995) while other report significant associations with emotion-focused coping strategies instead (e.g., David and Suls 1999). The inconsistent findings may explain the low effect sizes with burnout in previous studies. Thus, we expect teacher openness will be negatively associated with burnout (H2e).

## Moderators: Teacher Effectiveness Measure, Source of Report, and Educational Level

To fully appreciate the complexity of the associations between teacher personality and the two job-related outcomes, we investigate three possible moderators in our meta-analysis: (a) type of teacher effectiveness measure, (b) source of teacher personality report, and (c) level of education taught by the teacher. First, we examine whether the association between teacher personality and teacher effectiveness changes depending on the type of the teacher effectiveness measure (i.e., evaluations of teaching, student performance self-efficacy, classroom observations, and academic achievement). Since teacher effectiveness is multidimensional, it cannot be captured by a single criterion (Muijs 2006). Furthermore, the type of teacher effectiveness measure used may determine whether it is or is not associated with teacher personality. For example, a recent study found that teacher personality predicted student-reports of teacher academic support and teacher personal support but not academic achievement (Kim et al. 2018). A similar study conducted at tertiary education level found that teacher personality predicted student evaluations of teaching but not academic achievement (Kim and MacCann 2018). We expect that teacher personality will be associated with outcomes proximal to the predictor and high in fidelity (e.g., evaluations of teaching), but not with other measures distal to the predictor and low in fidelity (e.g., academic achievement; H3).

The difference between who one reports oneself to be (self-report) and who others report the individual to be (other-report) can lead to different strengths of empirical associations with the outcome variables. Self-reports are more easily prone to intentional and unintentional socially desirable responding, in order to hide socially undesirable qualities or to enhance desirable ones (Paulhus 2002). Such behavior can explain why meta-analytic findings that other-reports of an individual’s personality are stronger correlate with student academic achievement (Poropat 2014) and job performance (Connelly and Ones 2010; Oh et al. 2011) than self-reported personality. Similarly, we expect that teachers’ other-reported personality will be more strongly associated with the outcomes than teachers’ self-reported personality (H4).

Lastly, we examine whether teacher personality is important in different ways across educational levels (i.e., teachers teaching in elementary, secondary, and tertiary education). Poropat’s (2009) hypothesis on the reduced influence of student personality on academic achievement due to increased variety of learning environments and activities with increasing levels of education can also be applied in the teacher context. That is, at lower educational levels, teacher personality has a greater influence on a variety of outcomes, given the comparably limited sources of learning students may have. Furthermore, teachers may have more opportunities to give individual attention and foster student engagement, often as a result of smaller classes at lower education levels (Blatchford et al. 2011). Finally, the greater emphasis on delivering a centralized and structured curriculum at higher levels of education, that often requires self-directed learning (Wilcox 1996), may diminish the opportunity for teacher personality to be displayed in teaching. This difference may explain how teacher emotional stability predicted student performance self-efficacy in secondary school students (Kim et al. 2018) but not in tertiary education students (Kim and MacCann 2018). The effect of these three phenomena is that there are more opportunities for a teacher’s personality to be displayed and to be an influence to students. Thus, we expect that the association between teacher Big Five domains and the outcomes to be the strongest in lower educational levels (H5).

## Purpose of This Meta-Analysis

As previously mentioned, Klassen and Tze (2014) found a small though statistically significant association (*r* = .08, *p* < .05) between teacher personality and teacher effectiveness. However, there are three major limitations to the meta-analysis that may explain the small effect size. First, Klassen and Tze treated personality as a unidimensional construct by combining the multiple personality domains into one. Such an approach is problematic both theoretically and empirically. Of the very large number of theoretical models of personality, only one (the General Factor of Personality; Musek 2007) proposes that personality is a single dimension. Empirically, averaging multiple effect sizes from multiple domain can be problematic as large associations of one personality domain with the outcomes may be masked by small or negative effects from other personality domains. Second, Klassen and Tze combined results across multiple teacher effectiveness measures (i.e., evaluations of teaching and student academic achievement). Teacher effectiveness is a multidimensional construct, whereby one of its measures may not measure the same aspect as another (Muijs 2006). Thus, averaging across measures can mask the potential effect that personality has on one teacher effectiveness measure by another measure. Third, Klassen and Tze used restricted search terms, both in terms of predictors and outcome terms. As a result, their meta-analytic finding was the aggregated findings from only 10 studies.

Accordingly, we build upon Klassen and Tze’s (2014) findings in five primary ways. First, we examine personality as a multidimensional construct using the most established personality framework by considering each personality domains within the Big Five framework. Second, we examine multiple aspects of teacher effectiveness and their relationship with teacher personality as a moderator. Third, we aim to capture more studies in the meta-analysis by including more specific search terms for both the predictor and the outcomes. Fourth, we examine other moderators that could affect the association between teacher personality and outcomes (i.e., source of personality report and educational level). Fifth, we consider an additional important outcome within the teaching profession (teacher burnout).

In sum, this meta-analysis examines how teacher personality (using the Big Five framework) may be associated with teacher effectiveness and job burnout. Furthermore, three moderator effects will be examined: the effect of type of teacher effectiveness measure, source of personality report, and educational level.

## Method

### Literature Search

Our systematic literature search used three search strategies to identify all relevant literature concerning the relationship between teacher Big Five personality domains and the two job-related outcomes. First, we conducted an electronic search of the relevant articles using different databases relevant to our particular topic: PsycINFO (for psychological research and related fields), ERIC (for educational research), Web of Science (referencing science of all kinds), and Proquest Theses and Dissertations Global (in order to include unpublished literature and avoid publication bias). For our electronic search, we developed the following search term: (“teacher personality” OR “personality of teacher*” OR “teacher* disposition*” OR “disposition* of teacher*” OR “teacher* trait*” OR “trait* of teacher*” OR “teacher big five” OR “teacher five factor model” OR “teacher openness” OR “teacher intellect” OR “teacher agreeableness” OR “teacher conscientiousness” OR “teacher extraversion” OR “teacher emotional stability” OR “teacher neuroticism”) *AND* (*effectiveness OR grade OR performance OR achievement OR motivation OR commitment OR engagement OR satisfaction OR burnout OR retention OR attrition OR dropout OR outcome OR self-efficacy OR success OR health OR well-being OR efficacy*). We searched for articles with no date restrictions as well as both peer-reviewed and non-peer-reviewed articles to reduce the chance of including articles with publication biases. Second, we examined Klassen and Tze’s (2014) meta-analysis for studies they included on teacher personality and teacher effectiveness. Third, we examined studies citing Klassen and Tze’s meta-analysis.

**Fig. 1:**
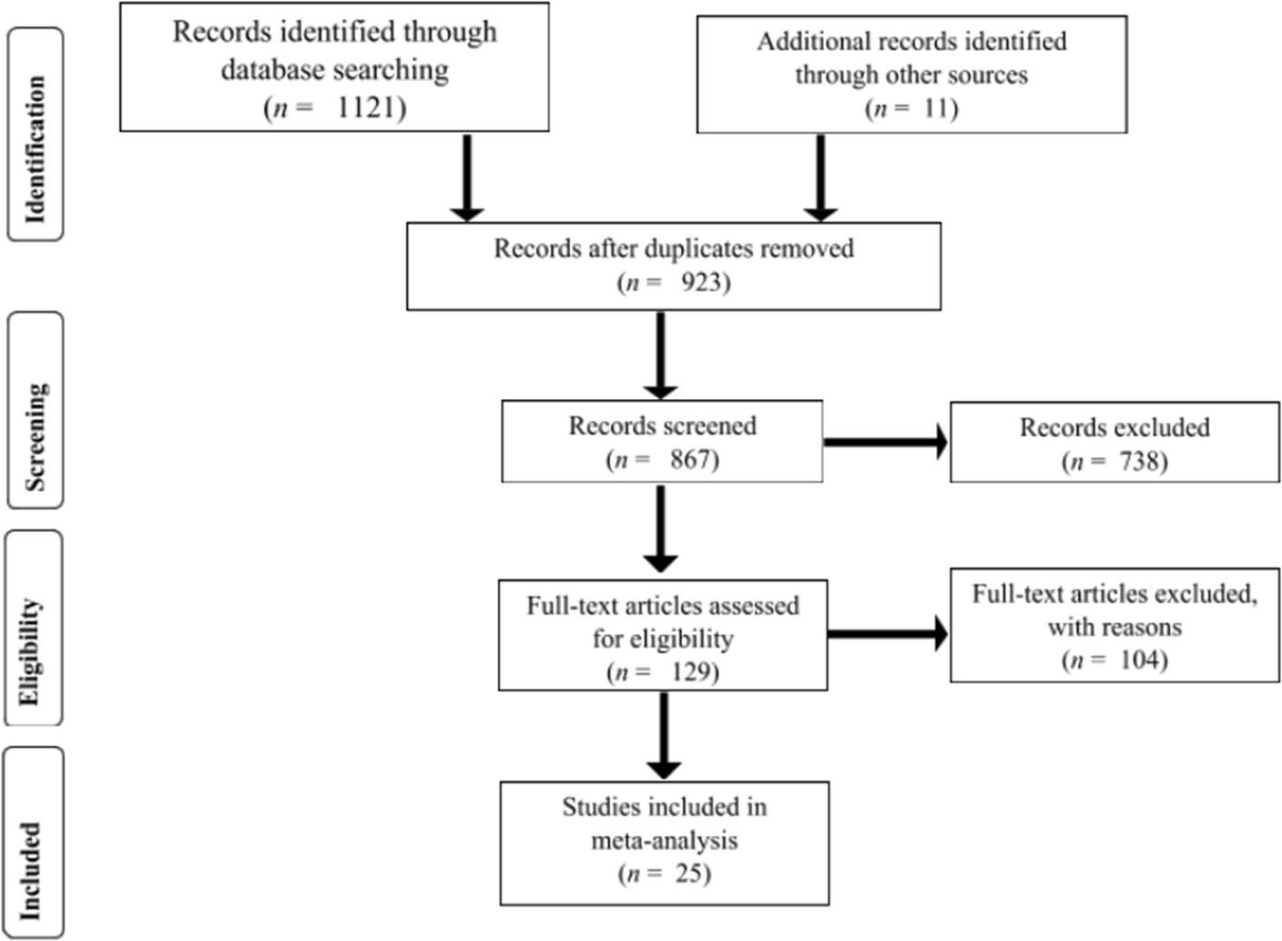
Flow chart of the inclusion and exclusion of studies

The electronic search resulted in a total of 1121 abstracts (PsycINFO 551 articles, Web of Science 90 articles, Proquest Dissertation Global 256 articles, ERIC 324 articles) and 11 additional studies. In the first step, we excluded 738 records. We excluded studies that were not applicable to the current meta-analysis, including expert opinions, theoretical discussions, and qualitative data. We also excluded studies reporting relationships not associated with our research questions, including studies on perspectives of students’ teacher personality preferences and on the associations between student personality and educational outcomes. This process led to further examining 129 studies. The flow diagram (see Fig. 1) illustrates our process through the different phases.

### Exclusion Criteria

We examined the remaining 129 studies by reading the full texts and excluded studies if a copy was not available (e.g., searching through the university open repositories) or if the study met one or more of these exclusion criteria:

- the personality measures could not be classified within the Big Five framework empirically or theoretically,
- the outcome measures could not be classified within the three criterion outcomes, and
- information on the statistical associations between the relevant variables was missing.

For example, we excluded studies on Myers-Briggs Type Indicator profiles of teachers because profiles cannot be transformed into levels within the Big Five domain. We also excluded studies not reporting effect sizes or descriptive statistics from which effect sizes could not be computed.

As per previous meta-analytic practices (e.g., Barrick and Mount 1991; Judge et al. 2002; Judge et al. 2013), we included studies with personality measures that could be classified within the Big Five framework. The process of classification involved examining the properties within the described characteristics and aligning them with the closest fitting Big Five domain, which were guided by previous works on Big Five classifications (e.g., Digman 1990; Goldberg 1999; Piedmont et al. 1992; Rossier et al. 2004). For example, the 16PF (Cattell et al. 1970) was aligned to the Big Five framework based on empirical strength associations studies (e.g., Rossier et al. 2004) and theoretical models (John et al. 2008). After applying the above-mentioned stages of inclusion and exclusion, 25 studies (18 studies with teacher effectiveness and 7 studies with teacher burnout) were included for final analysis.

### Calculating Effect Sizes

A program called Meta-Essentials (Suurmond et al. 2017) was used to conduct a random-effects meta-analysis. To compute the effect sizes for the overall effects and each moderator effect, individual Pearson *r* coefficients were transformed into Fisher’s *z* scores. When multiple Fisher’s *z* scores were present within one study (e.g., multiple effect sizes between teacher conscientiousness and multiple aspects of evaluations of teaching), the Fisher’s *z* scores were averaged within each study and by the type of moderator analysis category because including multiple effect sizes from the same study would violate the assumptions of independence (Lipsey and Wilson 2001). That is, mean effect sizes were calculated by averaging the Fisher’s *z* scores. The average Fisher’s *z* scores were then transformed back to Pearson *r* coefficients. These transformed Pearson *r* coefficients were then used to calculate the final effect sizes for the meta-analysis.

## Results

### Descriptive Statistics

A summary of all of the included studies is reported in Table 1. The majority of the studies (64% or 16/25) were PhD dissertations, with 32% (8/25) published in academic journals, and 4% (1/25) published as a report. Most of the studies—60%—were conducted in the USA (15/25), with the remaining studies conducted in Australia (2), Canada (2), Cyprus (1), Israel (1), Poland (1), Romania (1), Taiwan (1), and Turkey (1). The sample sizes ranged from 16 to 2671, *M* = 251.77, *SD* = 528.27.

**Table 1:** Summary of included studies

Study details					Big Five domains				Source of personality report				Educational level			
Authors and date	Type	Country	Personality measure	Outcome	Big Five domain	*N*	*k*	Effect size	Source	*N*	*k*	Effect size	Level	*N*	*k*	Effect size
Acaray and Yildirim (2017)	Dissertation	Turkey	NEO-PI (McCrae and Costa 1982)	Burnout	O	254	3	.07	S-R	254	3	.07	–	–		
					C	254	3	−.07		254	3	−.07				
					E	254	3	.03		254	3	.03				
					A	254	3	−.12		254	3	−.12				
					ES	254	3	.14		254	3	.14				
Chan (2003)	Dissertation	USA	BFI (John et al, 1991)	TE/EoT	O	44	25	.05	S-R	44	25	.05	Ter	44	25	.05
					C	44	25	.22		44	25	.22		44	25	.22
					E	44	25	.30		44	25	.30		44	25	.30
					A	44	25	.06		44	25	.06		44	25	.06
					ES	44	25	−.01		44	25	−.01		44	25	−.01
Chu (2003)	Dissertation	Taiwan	MPQ5 (Cameron 1996)	TE/EoT	O	130	1	.11	S-R	130	1	.11	Ele	130	1	.11
					C	130	1	.28		130	1	.28		130	1	.28
					E	130	1	.12		130	1	.12		130	1	.12
					A	130	1	−.17		130	1	−.17		130	1	−.17
					ES	130	1	.19		130	1	.19		130	1	.19
Colomeischi (2015)	Journal article	Romania	FFPI (Hendriks et al. 1999)	Burnout	O	575	3	.23	S-R	575	3	.23	–	–		
					C	575	3	.32		575	3	.32				
					E	575	3	.32		575	3	.32				
					A	575	3	.28		575	3	.28				
					ES	575	3	.38		575	3	.38				
					E	188	4	.27		188	4	.27				
					A	188	2	.29		188	2	.29				
					ES	188	3	.37		188	3	.37				
Cooper and Bemis (1967)	Report	USA	ToPS (self-developed)	TE	O	60	27	−.06	O-R	60	27	−.06	Ele	60	27	−.06
				CO			7	−.07								
				AA			20	−.06								
				TE	C	60	25	.01		60	25	.01		60	25	.01
				CO			15	.02								
				AA			10	−.01								
				TE	E	60	30	.01		60	30	.01		60	30	.01
				CO			10	.01								
				AA			20	.01								
				TE	A	60	17	.09		60	17	.09		60	17	.09
				CO			7	.11								
				AA			10	.07								
				TE	ES	60	17	−.07		60	17	−.07		60	17	−.07
				CO			7	−.01								
				AA			10	−.13								
Cutchin (1998)	Dissertation	USA	NEO-PI-R (Costa and McCrae 1992)	TE/EoT	O	123	6	.06	S-R	123	6	.06	Sec	123	6	.06
					C	123	6	.09		123	6	.09		123	6	.09
					E	123	6	.04		123	6	.04		123	6	.04
					A	123	6	−.07		123	6	−.07		123	6	−.07
					ES	123	6	.01		123	6	.01		123	6	.01
Emmerich et al. (2006)	Journal article	USA	AFSDI (Christal 1994; Tupes and Christal 1992)	TE/CO	O	2671	1	.03	S-R	2671	1	.03	–	–		
					C	2671	1	.01		2671	1	.01				
					E	2671	1	.00		2671	1	.00				
					A	2671	1	−.09		2671	1	−.09				
					ES	2671	1	−.05		2671	1	−.05				
Isaacson et al. (1963)	Journal article	USA	16 PF (Cattell et al. 1970)	TE/EoT	O	16	19	.17	S-R	16	19	.17	Ter	16	19	.17
					C	16	12	−.33		16	12	−.33		16	12	−.33
					E	16	16	.15		16	16	.15		16	16	.15
					A	16	12	.17		16	12	.17		16	12	.17
					ES	16	24	−.10		16	24	−.10		16	24	−.10
Jamil et al. (2012)	Journal article	USA	NEO-FFI (Costa and McCrae 1992)	TE/Co	E	509	3	.02	S-R	509	3	.02	–	–		
					ES	509	3	.04		509	3	.04				
Job (2004)	Dissertation	USA	NEO-FFI (Costa and McCrae 1992)	TE/EoT	O	190	1	−.04	S-R	190	1	−.04	Ter	190	1	−.04
					C	190	1	.03		190	1	.03		190	1	.03
					E	190	1	.08		190	1	.08		190	1	.08
					A	190	1	−.04		190	1	−.04		190	1	−.04
					ES	190	1	.10		190	1	.10		190	1	.10
Jones (2014)	Dissertation	USA	NEO-FFI-3 (McCrae and Costa 2010)	TE/CO	O	57	7	.10	S-R	57	7	.10	Ele	57	7	.10
					C	57	7	.07		57	7	.07		57	7	.07
					E	57	7	.10		57	7	.10		57	7	.10
					A	57	7	.03		57	7	.03		57	7	.03
					ES	57	7	.13		57	7	.13		57	7	.13
Kim et al. (2018)	Journal article	Australia	BFI (John, 1990)	TE	O	54.83	24	.26	O-R	89	8	.51	Sec	54.8	24	.26
				EoT		55	12	.42								
				SPS		54.33	6	.06	S-R	37.8	16	.12				
				AA		55	6	.11								
				TE	C	54.83	24	.20	O-R	89	8	.45		54.8	24	.20
				EoT		55	12	.32								
				SPS		54.33	6	.11	S-R	37.8	16	.05				
				AA		55	6	.02								
				TE	E	54.83	24	.24	O-R	89	8	.43		54.8	24	.24
				EoT		55	12	.34								
				SPS		54.33	6	.13	S-R	37.8	16	.13				
				AA		55	6	.13								
				TE	A	54.83	24	.29	O-R	89	8	.51		54.8	24	.29
				EoT		55	12	.45								
				SPS		54.33	6	.11	S-R	37.8	16	.16				
				AA		55	6	.09								
				TE	ES	54.83	24	.22	O-R	89	8	.42		54.8	24	.22
				EoT		55	12	.33								
				SPS		54.33	6	.18	S-R	37.8	16	.11				
				AA		55	6	.03								
Kim & MacCann (2018)	Journal article	Australia	Mini-Markers (Saucier 1994)	TE	O	66.5	18	.13	O-R	88	9	.30	Ter	66.5	18	.13
				EoT		66.5	14	.17								
				SPS		66.5	2	.10	S-R	45	9	−.04				
				AA		66.5	2	−.07								
				TE	C	66.5	18	.14	O-R	88	9	.34		66.5	18	.14
				EoT		66.5	14	.20								
				SPS		66.5	2	−.15	S-R	45	9	−.07				
				AA		66.5	2	−.05								
				TE	E	66.5	18	.15	O-R	88	9	.39		66.5	18	.15
				EoT		66.5	14	.21								
				SPS		66.5	2	−.08	S-R	45	9	−.11				
				AA		66.5	2	−.08								
				TE	A	66.5	18	.16	O-R	88	9	.37		66.5	18	.16
				EoT		66.5	14	.22								
				SPS		66.5	2	−.10	S-R	45	9	−.07				
				AA		66.5	2	−.07								
				TE	ES	66.5	18	.08	O-R	88	9	.33		66.5	18	.08
				EoT		66.5	14	.11								
				SPS		66.5	2	−.14	S-R	45	9	−.19				
				AA		66.5	2	.03								
Klis (1997)	Journal article	Poland	SEE (Mehrabian and Epstein 1972)	Burnout	A	130	24	−.01	S-R	130	24	−.01	Ele	130	24	−.01
Kokkinos (2007)	Journal article	Cyprus	NEO-FFI (Costa and McCrae 1992)	Burnout	O	447	3	.02	S-R	447	3	.02	Ele	447	3	.02
					C	447	3	.07		447	3	.07		447	3	.07
					E	447	3	−.04		447	3	−.04		447	3	−.04
					ES	447	3	−.19		447	3	−.19		447	3	−.19
Monsour (1987)	Dissertation	USA	CPI (Megargee 1972)	TE/CO	A	48	1	.00	S-R	48	1	.00	Ele	48	1	.00
Murray (1975)	Journal article	Canada	20 personality traits (self-developed)	TE/EoT	O	36	1	.59	O-R	36	1	.59	Ter	36	1	.59
					C	36	1	.39		36	1	.39		36	1	.39
					E	36	5	.49		36	5	.49		36	5	.49
					A	36	2	.07		36	2	.07		36	2	.07
					ES	36	2	.58		36	2	.58		36	2	.58
Murray et al. (1990)	Journal article	Canada	JPRF (1984) & EPQ (Eysenck and Eysenck 1975)	TE/EoT	O	46	31	.18	O-R	46	31	.18	Ter	46	31	.18
					C	46	33	.26		46	33	.26		46	33	.26
					E	46	43	.35		46	43	.35		46	43	.35
					A	46	30	.09		46	30	.09		46	30	.09
					ES	46	41	−.02		46	41	−.02		46	41	−.02
Phillips et al. (1985)	Journal article	USA	16 PF (Cattell et al. 1970)	TE	O	18	12	.05	S-R	18	12	.05	–	–		
				CO		18	10	.01								
				AA		18	2	.20								
				TE	C	18	12	−.08		18	12	−.08				
				CO		18	10	−.04								
				AA		18	2	−.31								
				TE	E	18	24	−.01		18	24	−.01				
				CO		18	19	−.04								
				AA		18	5	.14								
				TE	A	18	12	−.13		18	12	−.13				
				CO		18	10	−.12								
				AA		18	2	−.17								
				TE	ES	18	24	.03		18	24	.03				
				CO		18	19	.02								
				AA		18	5	.11								
Radmacher and Martin (2001)	Journal article	USA	SEFF (self-developed)	TE	E	348	2	.59	O-R	348	2	.59	Ter	348	2	.59
				EoT		348	1	.79		348	1	.79		348	1	.79
				AA		348	1	.28		348	1	.28		348	1	.28
Richard (1992)	Dissertation	USA	EPQ (Eysenck and Eysenck 1975)	TE/AA	E	44	9	.01	S-R	44	9	.01	Ele	44	9	.01
					ES	44	10	.16		44	10	.16		44	10	.16
Ripski et al. (2011)	Journal article	USA	NEO-FFI (Costa and McCrae 1992)	Burnout	O	67	3	−.21	S-R	67	3	−.21	–	–		
					C	67	3	.29		67	3	.29				
					E	67	3	.30		67	3	.30				
					A	67	3	.28		67	3	.28				
					ES	67	3	.51		67	3	.51				
Shapiro (1987)	Dissertation	USA	16 PF (Cattell et al. 1970)	Burnout	E	220	1	−.04	S-R	220	1	−.04	Ele	220	1	−.04
					ES	220	1	.00		220	1	.00		220	1	.00
Shechtman (1989)	Journal article	Israel	16 PF (Cattell et al. 1970)	TE/CO	O	92	3	.20	O-R	92	2	.18	–	–		
									S-R	92	1	.24				
					C	92	1	.28	O-R	92	1	.28				
					E	92	4	.18	O-R	92	2	.24				
									S-R	92	2	.13				
					A	92	2	.33	O-R	92	1	.42				
									S-R	92	1	.24				
					ES	92	2	.34	O-R	92	2	.34				
Teven (2007)	Journal article	USA	Big Five measure (Sager and Gastil 2002)	Burnout	O	48	3	−.09	S-R	48	3	−.09	Ter	48	3	−.09
					C	48	3	.39		48	3	.39		48	3	.39
					E	48	3	.28		48	3	.28		48	3	.28
					A	48	3	.28		48	3	.28		48	3	.28
					ES	48	3	.42		48	3	.42		48	3	.42

*Note. k* = number of effect sizes; EoT = evaluations of teaching; SPS = student performance self-efficacy; CO = classroom observation; AA = academic achievement; NEO-PI = NEO Personality Inventory; BFI = Big Five Inventory; MPQ5 = Manchester Personality Questionnaire 5; FFPI = Five-Factor Personality Inventory; ToPS = Teacher Observation Personality Schedule; NEO-PI-R, NEO Personality Inventory Revised; AFSDI = Air Force Self-description Inventory; 16 PF = 16 Personality Factors; NEO-FFI = NEO Five Factory Inventory; SEE = Scale of Emotional Empathy; CPI = California Psychological Inventory; JPRF, Jackson’s Personality Research Form; EPQ = Eysenck Personality Questionnaire; SEEF = Student Evaluation of Faculty Forum; O = openness; C = conscientiousness; E = extraversion; A = agreeableness; ES = emotional stability; S-R = self-rated; O-R, other-rated; Ele = elementary; Sec = secondary; Ter = tertiary

### Overall Effects

The associations between each of the criterion outcomes with the Big Five domains were examined. Estimates of the overall effect as well as the three moderators can be found in Table 2, outlining the corresponding number of effect sizes (*k*), effect sizes, 95% lower and upper confidence intervals, *Q-statistic* with *p* value, and *I*^2^.

**Table 2:** Correlations between teacher Big Five personality and job-related outcomes, and moderators (teacher effectiveness measure, source of personality report, and educational level)

Moderator	*k*	Effect size	95% CI (LL, UL)	*Q*	*p*_Q_	*I*^2^ (%)
Job-related outcomes						
Openness						
Teacher effectiveness	14	0.10	(0.01, 0.18)	22.76	0.04	42.87
EoT	9	0.17	(0.01, 0.33)	21.62	0.01	63.00
SPS	2	0.09	(− 0.18, 0.34)	0.05	0.82	0.00
CO	5	0.03	(− 0.01, 0.08)	3.38	0.50	0.00
AA	4	0.01	(− 0.17, 0.18)	1.70	0.64	0.00
Burnout	5	0.04	(− 0.16, 0.23)	20.53	0.00	80.52
Conscientiousness						
Teacher effectiveness	14	0.13	(0.04, 0.21)	28.11	0.01	53.75
EoT	8	0.19	(0.08, 0.29)	10.03	0.19	30.24
SPS	2	0.02	(− 0.75, 0.77)	0.70	0.40	0.00
CO	5	0.07	(− 0.08, 0.22)	6.85	0.14	41.58
AA	4	−0.07	(− 0.26, 0.12)	2.04	0.56	0.00
Burnout	5	0.19	(− 0.06, 0.41)	37.22	0.00	89.25
Extraversion						
Teacher effectiveness	17	0.17	(0.07, 0.27)	159.16	0.00	89.95
AA	6	0.10	(− 0.06, 0.25)	11.31	0.05	55.80
CO	6	0.01	(− 0.03, 0.05)	3.55	0.62	0.00
EoT	10	0.32	(0.10, 0.50)	199.12	0.00	95.48
SPS	2	0.02	(− 0.88, 0.89)	1.36	0.24	26.39
Burnout	6	0.13	(− 0.06, 0.31)	46.92	0.00	89.34
Agreeableness						
Teacher effectiveness	15	0.03	(− 0.05, 0.12)	33.14	0.00	57.76
EoT	9	0.07	(− 0.09, 0.22)	21.17	0.01	62.21
SPS	2	0.00	(− 0.87, 0.87)	1.25	0.26	20.02
CO	6	0.06	(− 0.13, 0.23)	19.28	0.00	74.07
AA	4	0.01	(− 0.15, 0.16)	1.35	0.72	0.00
Burnout	5	0.13	(− 0.11, 0.37)	34.35	0.00	88.35
Emotional stability						
Teacher effectiveness	16	0.10	(0.01, 0.18)	44.63	0.00	66.39
EoT	10	0.13	(− 0.01, 0.26)	16.46	0.04	51.39
SPS	2	0.01	(− 0.97, 0.97)	2.99	0.08	66.51
CO	6	0.06	(− 0.09, 0.21)	17.42	0.00	71.29
AA	5	0.02	(− 0.12, 0.15)	2.20	0.70	0.00
Burnout	6	0.21	(− 0.09, 0.48)	107.14	0.00	95.33
Source of personality report						
Openness	22	0.16	(− 0.09, 0.38)	72.62	0.00	71.08
Other-reported	6	0.29	(0.02, 0.52)	20.09	0.00	75.12
Self-reported	16	0.06	(0.00, 0.12)	31.12	0.01	51.80
Conscientiousness	22	0.20	(0.00, 0.37)	101.61	0.00	80.32
Other-reported	6	0.29	(0.12, 0.44)	8.90	0.11	43.83
Self-reported	15	0.11	(0.02, 0.20)	72.93	0.00	80.80
Extraversion	26	0.23	(− 0.08, 0.50)	229.41	0.00	89.10
Other-reported	7	0.37	(0.19, 0.53)	32.32	0.00	81.43
Self-reported	19	0.09	(0.02, 0.15)	69.10	0.00	73.95
Agreeableness	22	0.16	(− 0.11, 0.40)	151.06	0.00	86.10
Other-reported	6	0.29	(0.07, 0.48)	14.17	0.01	64.71
Self-reported	16	0.04	(− 0.05, 0.13)	90.82	0.00	83.48
Emotional stability	23	0.17	(− 0.01, 0.32)	198.25	0.00	88.90
Other-reported	6	0.28	(0.00, 0.51)	19.02	0.00	73.71
Self-reported	17	0.10	(0.00, 0.21)	153.91	0.00	89.60
Educational level						
Openness	13	0.05	(− 0.01, 0.10)	20.91	0.05	42.62
Elementary	4	0.03	(− 0.05, 0.12)	1.57	0.67	0.00
Secondary	2	0.13	(− 0.82, 0.89)	1.59	0.21	37.28
Tertiary	7	0.13	(− 0.10, 0.35)	16.82	0.01	64.33
Conscientiousness	13	0.13	(0.09, 0.17)	18.10	0.11	33.72
Elementary	4	0.12	(− 0.07, 0.30)	5.23	0.16	42.67
Secondary	2	0.12	(− 0.48, 0.65)	0.45	0.50	0.00
Tertiary	7	0.18	(− 0.01, 0.36)	11.95	0.06	49.78
Extraversion	16	0.14	(− 0.07, 0.34)	132.87	0.00	88.71
Elementary	6	0.00	(− 0.07, 0.06)	3.39	0.64	0.00
Secondary	2	0.11	(− 0.82, 0.88)	1.52	0.22	34.04
Tertiary	8	0.31	(0.14, 0.46)	52.82	0.00	86.75
Agreeableness	13	0.03	(− 0.05, 0.11)	15.64	0.21	23.29
Elementary	4	− 0.05	(− 0.24, 0.15)	3.40	0.33	11.82
Secondary	2	0.10	(− 0.98, 0.98)	4.89	0.03	79.55
Tertiary	7	0.06	(− 0.05, 0.17)	5.00	0.54	0.00
Emotional stability	15	0.07	(− 0.03, 0.16)	52.01	0.00	70.45
Elementary	6	0.02	(− 0.14, 0.18)	20.66	0.00	75.79
Secondary	2	0.09	(− 0.84, 0.89)	1.64	0.20	38.97
Tertiary	7	0.17	(− 0.07, 0.39)	16.79	0.01	64.26

*Note. k,* number of effect sizes; 95% CI (LL, UL), 95% confidence interval (lower limit, upper limit); *Q*, *Q*-statistic, *p*_Q_, *p* value for the *Q*-statistic; EoT, evaluations of teaching; SPS, student performance self-efficacy; CO, classroom observation; AA, academic achievement

### Teacher Effectiveness

For teacher effectiveness (*k* = 14 to 17), all Big Five domains, except for agreeableness, were significant correlates, with the greatest effect sizes for extraversion (*r* = .17, *p* < .05), conscientiousness (*r* = .13, *p* < .05), emotional stability (*r* = .10, *p* < .05), openness (*r* = .10, *p* < .05), and lastly agreeableness (*r* = .03, *p* > .05). Thus, the results were in support of H1a-e, but not H1d.

In regard to the heterogeneity of the effects, the *Q-statistic* (or Cochrane’s *Q*) was significant for each of the Big Five domains, *Q*(14 to 17) = 22.76 to 159.16, *p* < .001, indicating that there were significantly different effect sizes across studies. Furthermore, *I*^2^s ranged from 42.87 to 89.95%, indicating that there were substantial proportions of variability across the studies. These two measures indicated the suitability of moderator analyses.

### Burnout

As the numbers of effect sizes for burnout were very small (*k* = 5 to 6), the results should be approached with caution. The small number of effect sizes may also explain why none of the effects were statistically significant. Nevertheless, the greatest effect sizes were for emotional stability (*r* = .21, *p* > .05), conscientiousness (*r* = .19, *p* > .05), extraversion (*r* = .13, *p* > .05), agreeableness (*r* = .13, *p* > .05), and lastly openness (*r* = .04, *p* > .05). In terms of relative effect sizes, H2a-e were supported.

### Publication Bias

Three indicators were used to assess publication bias in the estimates using a fixed estimates model per Big Five domain. The two criterion outcomes were combined for this purpose given the small number of effect sizes in some of the criterion outcomes. First, the relatively symmetrical spread of the estimates in the funnel plots for each Big Five domain with the combined criterion outcomes indicate that there may not be a publication bias. Second, the non-significant Egger’s regression test (Egger et al. 1997) with an intercept estimate of 0.54 (95% CI − 0.62 to 1.70) for openness, 1.18 (95% CI − 0.30 to 2.66) for conscientiousness, 1.46 (95% CI − 0.62, 3.53) for extraversion, 1.35 (95% CI − 0.18, 2.88) for agreeableness, and 1.79 (95% CI − 0.10, 3.67) for emotional stability. Finally, Rosenthal’s (1979) fail-safe *N* tests indicate that a large number of studies (i.e., 113, 280, 589, and 258 studies for openness, conscientiousness, extraversion, and emotional stability, respectively) with non-significant results would be needed to make the association non-significant, which again indicates that there may not be a publication bias, except for agreeableness (*n* = 18). Thus, there did not appear to be major publication bias in the findings.

### Moderated Effects

To obtain a more nuanced understanding of the effect of teacher personality on the two job-related outcomes, three moderator analyses were conducted. In regard to the type of teacher effectiveness measure as a moderator (*k* = 19 to 24), only evaluation of teaching was significantly associated with teacher personality, specifically with three domains: openness (*r* = .17, *p* < .05), conscientiousness (*r* = .19, *p* < .05), and extraversion (*r* = .32, *p* < .05). Across the Big Five domains, the student academic achievement correlation was the smallest and was also non-significant. Overall, in support of H3, the strongest association between teacher personality and the teacher effectiveness outcomes was evaluations of teaching and the weakest was academic achievement.

The associations between the two sources of teacher personality report (self-report vs other-report) and the outcomes were examined. Other-reported teacher personality (*k* = 6 to 7) were consistently and moderately-strongly associated with the outcomes in general (*r* = .28 to .37), which were all statistically significant except for emotional stability. On the other hand, self-reported teacher personality (*k* = 15 to 19) were consistently but weakly associated with the outcomes in general (*r* = .04 to .11), of which only conscientiousness and extraversion were statistically significant. Overall, the correlations for other-reports were consistently stronger than self-reports for each Big Five domain (*z* = 2.15 to 3.50, *p* < .05), except for emotional stability (*z* = 1.48, *p* > .05), thus indicating support for H4.

The association between the three educational levels the teacher instructs in (i.e., elementary, secondary, and tertiary) and the outcomes in general were examined. Teacher personality at elementary (*k* = 4 to 6), secondary (*k* = 2), and tertiary level (*k* = 7 to 8) were not statistically associated with the outcomes in general (*r* = −.05 to .12, *r* = .09 to .13, *r* = .06 to .18, *p* > .05, respectively). The exception was extraversion at tertiary level, which was moderately associated with the outcomes in general (*r* = .31, *p* < .05). There were no differences between elementary and secondary levels (*z* = 0.68 to 1.42, *p* > .05) nor between secondary and tertiary levels for each Big Five domain (*z* = 0.02 to 0.67, *p* > .05). The exception was that extraversion at tertiary level was stronger than at secondary level (*z* = 1.73, *p* < .05), which indicated little support for H5.

## Discussion

In response to increasing attention on understanding the teacher personal characteristics associated with job-related outcomes (e.g., Sutcher et al. 2016), various meta-analyses on teacher factors have been conducted (e.g., Brown 2012; Mérida-López and Extremera 2017; Visible Learning Plus 2018). Teacher personality has also been the subject of similar investigations; however, none of the studies were a meta-analysis conceptualizing teacher personality as a multidimensional construct. Accordingly, the current meta-analysis investigated the association between teacher personality (within the Big Five framework) and teacher effectiveness and teacher burnout.

### Job-Related Outcomes

Teacher Big Five domains indeed have important links with teacher effectiveness. Visible Learning Plus (2018) reported that teacher personality is associated with student learning and Klassen and Tze (2014) similarly reported that teacher personality is associated with teacher effectiveness (a sum of student achievement and evaluated teaching performance). However, it was impossible to dissect which elements of a teacher’s personality was important given their unidimensional conceptualization of teacher personality. We found in our study that four of the Big Five domains (not agreeableness) can shape students’ educational experiences and outcomes. Teachers’ levels of extraversion and conscientiousness were particularly important, especially for student evaluations of teaching. Meta-analyses summarizing findings from multiple occupations report that conscientiousness is the strongest predictor of job performance (Barrick and Mount 1991; Salgado 2003). However, we found that extraversion was slightly more strongly associated with teacher effectiveness than conscientiousness. The fundamental social nature of the teaching profession may explain this finding, whereby one’s level of assertiveness and energy is just as important or even more important than one’s level of achievement-focus and organization. This finding seems to indicate that job performance in organizations may not be equivalent to job performance in the teaching profession. Providing instructional and emotional support are primary components of the teaching profession (Danielson 2013; Pianta et al. 2008), but these are not typical markers of job performance in other organizations, which can thus help explain why extraversion was a stronger predictor than conscientiousness.

Contrary to expectation, agreeableness was not associated with teacher effectiveness. This finding was surprising as a meta-analysis found that this domain was more important for jobs requiring teamwork (and, therefore, greater interpersonal interactions) than those involving dyadic interactions (Mount et al. 1998). However, our finding may be an artifact of examining only one profession, whereby the limited variation of agreeableness across teachers may have caused a restricted statistical correlation with the outcome. Mount et al. (1998) compared the job performance of employees in manufacturing plants working in teams (teamwork group) against employees from a variety of settings (dyadic service group). Given the limited specificity of the inclusion criteria of the teamwork group, the samples may have been from different jobs and be different types of people, which in effect would result in large variance within the teamwork group and thus a significant finding. In contrast, our meta-analysis included only individuals in the teaching profession. As further evidence to the possibility that our finding may be a statistical artifact, a meta-analysis of meta-analyses found that the effect sizes between agreeableness within occupation groups (i.e., sales, managers, professionals, police, and skilled or semi-skilled labor) and job performance were close to zero and non-significant for two groups (i.e., sales and skilled or semi-skilled laborers; Barrick et al. 2001). Thus, we warn against drawing practical implications from this finding.

In regard to teacher burnout, although Chang (2009) identified teacher personality characteristics were associated with burnout, she did not identify the Big Five domains. Studies from across multiple professions have found though that burnout was most strongly associated with emotional stability, followed by conscientiousness and extraversion (Alarcon et al. 2009; Swider and Zimmerman 2010). Similar to their findings, we found that in terms of effect sizes, teachers’ low levels of emotional stability were most strongly associated with burnout followed by high levels of conscientiousness, extraversion, agreeableness, and openness. However, these effects were not statistically significant, which may be due to the small number of effect sizes in the analysis. Thus, more studies should be conducted on the possible association between teacher personality and burnout before these findings can be generalized.

### Moderators: Teacher Effectiveness Measure, Source of Report, and Educational Level

Multiple types of teacher effectiveness measures are available (see Goe et al. 2008 for a review) and so we examined this factor as a potential moderator. Evaluation of teaching was the only type of teacher effectiveness measure associated with teacher personality, namely with extraversion, conscientiousness, and openness. The majority of the evaluations of teaching in the meta-analysis were in the form of student evaluations of teaching, indicating that students are affected by the teachers’ display of their levels of energy, organization skills, and intellectual curiosity in the classroom and in school in general. These three personality domains may be important because these qualities are often featured in student evaluations of teaching. For example, Marsh’s (1984) Student Evaluations of Educational Quality questionnaire contains seven domains assessing the level of university teaching, including group interaction (stimulation of classroom discussions and enquiry), enthusiasm (level of enthusiasm in the classroom), and organization (clarity of instructions, preparation of course material). These three teaching domains are strongly associated with teacher openness, extraversion, and conscientiousness, respectively (Kim and MacCann 2018). Our results highlight the need to consider the impact of teacher personality on student educational experiences, which can often be overseen in educational research.

Obtaining self-reported personality is often logistically easier than other-reported personality. However, other-reports are sometimes better predictors than self-reports due to multiple factors, such as one’s tendency to fake in self-assessments and having limited self-knowledge (see Vazire and Carlson 2011 for a review). As personality questionnaires are often very transparent on what the socially desirable answers are, individuals can fake their responses, whereby they consciously or unconsciously distort their responses in a way that enhances their positive characteristics and suppresses their negative characteristics (McFarland and Ryan 2000). For example, a study found that teacher education students’ responses to personality questionnaires were different before the application stage of the program and 6 months afterwards, indicating that they had potentially altered their personality responses to reflect socially desirable responses (Krammer et al. 2017). Thus, we assessed source of report as a moderator, given that self-reports can contain distorted responses. Other-reports of teacher personality indeed were more strongly related to job-related outcomes than self-reports, except for emotional stability, where the effect was stronger for other-reports but not statistically significantly so. Overall, our findings were consistent with meta-analytic findings that other-reports have higher validities than self-reports (Connelly and Ones 2010; Oh et al. 2011; Poropat 2014). Thus, it seems that assessing personality from another person’s perspective may be useful in teacher research as it is in other fields.

The teacher personality–outcome effect sizes across the educational levels seemed to grow with higher levels of education, although the finding was not statistically significant. For example, the effect of openness was particularly prominent in secondary and tertiary levels. The effect seemed to also increase for conscientiousness, extraversion, and emotional stability, which was contrary to expectation. Longitudinal studies have found that individuals change in their level of personality and the most personality trait change occurs during young adulthood (Roberts et al. 2006). Thus, students may be looking for and most benefit from people who are similar to them at different stages of their lives, as a match between student and teacher personality can be beneficial for student educational experiences (Kim and MacCann 2016).

### Theoretical and Practical Implications of Findings

Understanding the personal characteristics of effective teachers through a consensual evidence-based framework, such as the Big Five framework, can help in theory development and refinement. Multiple descriptive frameworks are used by educational researchers to describe the qualities of effective teachers. Yet, there are no consensual models nor terminologies that group these descriptors together. Using common descriptors and models is helpful when conducting and disseminating research. Personality models have been established and tested for decades, including the Big Five framework, for the purpose of giving a common language to describing human characteristics (John et al. 2008). Using common descriptors can help us identify the predictors of important outcomes and later identify the mediators and moderators of such relationships. As such, theoretical models outlining the links between teacher personal characteristics, the processes, and the relevant outcomes can be established by using descriptors within a consensual framework. We agree with Göncz (2017) that findings from studies that use established personality theories, such as our current meta-analysis, can inform the building of a comprehensive theory of teacher personality within an educational psychology context.

Before practical implications can be suggested, one should be aware of the current educational climate. The use of some assessments for making high-stake decisions has ignited a controversial debate among practitioners and policymakers. For example, some states in the USA are using student academic achievement outcomes to make high-stake decisions about teachers (e.g., salary, retention, and hiring), which has provoked the American Educational Research Association (AERA) to release a statement on how these evaluations should and should not be used (American Educational Research Association 2015). In line with AERA’s views and being respective of the sensitive climate around assessment, we suggest that personality assessments should not be used for selection but could be used for professional feedback purposes.

Organizations have for decades used personality assessments to inform personnel selection decisions (see Salgado and De Fruyt 2017 for a review). The usefulness of personality assessments to aid in selection in the teaching arena is, thus, an inevitable topic of discussion. Researchers have previously suggested using personality assessments to aid in selection decisions for initial teacher education programs (Thornton et al. 2005) and for entering teaching practice (Kennedy 2008). As our meta-analysis shows, there are a limited number of studies on teacher personality and its associations with important outcomes (e.g., teacher effectiveness). Furthermore, it is unclear whether the standard conceptualizations and phenomena drawn from and found in organizational psychology can be directly applied to educational systems and institutions. That is, given the unique nature of the educational context and the teaching profession, it is questionable whether policies and practices used in organizations can be directly transferred and applied in the teaching profession. Thus, using personality assessments to inform selection decisions for pre-service and in-service teachers may be premature in its current state.

The debate whether personality is stable or malleable has been continuing for decades (e.g., McCrae and Costa 1982). There is growing evidence, however, that personality can change although very modestly through age (e.g., see Roberts et al. 2006 for a review) and also through interventions (see Roberts et al. 2017 for a review). Moreover, the way that others perceive an individual can differ from how one perceives themselves, including in personality assessment (Vazire 2010). In light of this evidence, organizations often conduct multisource or 360-degree feedback. This feedback is a reflective assessment of how an employee perceive themselves, is perceived by their superior, and their fellow colleagues (DeNisi and Kluger 2000), which can help improve future job performance (Smither et al. 2005). Accordingly, personality assessments could provide an individual feedback about which qualities they may wish to enhance or suppress depending on the context. Namely, such feedback can help teachers reflect on how they may be perceived at school, how they may perceive themselves, and the potential impact they may be having on the school environment and individuals. As a result, their self-awareness may increase, and they may choose to engage in a form of skill development.

Interventions to increase students’ social and emotional skills are increasingly popular, with promising evidence of their effectiveness (Durlak et al. 2011). Similar programs could be offered to teachers as part of their continued professional development, especially given that the effectiveness of current interventions for reducing teacher burnout is minimal (see Iancu et al. 2018 for a review). One example may be offering emotion regulation workshops to teachers. Emotional stability is often associated with difficulties in regulating one’s emotions (see Segerstrom and Smith 2019 for a review), which can have negative influences on teachers (e.g., emotional exhaustion; Tsouloupas et al. 2010). As such, personality assessments could be beneficial for the teacher (e.g., well-being) but also for the students and the school in general.

### Limitations and Future Directions

The results from the meta-analysis, especially the moderator analyses, should be approached with caution. Some of the studies contained small sample sizes, which could have compromised the quality of data. Furthermore, the small number of effect sizes within each category may have contributed to the non-significant findings. Nevertheless, the magnitude of the effect sizes is a promising indicator, encouraging further studies of the associations between teacher personality and burnout as well as its moderators.

Furthermore, most of the studies included in the meta-analysis were from countries that could be classified as Western, Educated, Industrialized, Rich, and Democratic (WEIRD). Increasingly, researchers are recognizing that collecting samples from WEIRD samples and generalizing the findings to non-WEIRD populations are strongly questionable (Henrich et al. 2010). Given that norms of teacher practices, student behaviors, and educational contexts often differ between countries (Klassen et al. 2018), it is important that future studies on teacher personality are conducted in a variety of countries.

Finer-grained examinations of the link between teacher personality and outcomes could also be investigated with further research on this topic. Examining teacher Big Five at the facet level could provide further information on which elements of teachers’ personality are important (see Soto and John 2017 for a discussion on personality domain and facet measurement). An alternative approach is to examine teacher personality using different personality models. For example, the HEXACO model—with six domains of honesty-humility, extraversion, agreeableness, conscientiousness, and openness—has empirical, theoretical, and practical advantages over the Big Five framework (see Ashton and Lee 2007 for a review), which could be worthy of study in teacher personality research. As such, more studies should be conducted in this area to strengthen the generalizability of the findings as researchers explore more of the mechanisms between teacher personality and important outcomes.

Our meta-analysis was a cross-sectional analysis, whereby the correlation between teacher personality and an outcome measure given at a certain time was examined. However, some researchers found that teacher effects carry over time, including the likelihood of students dropping out, taking SATs, intending to go to college, attending higher-ranked collages, earning higher salaries, living in higher SES neighborhoods, and having more retirement savings (Chetty et al. 2011; Judge et al. 2013). Given the limited number of studies in our meta-analysis, such examination could not be undertaken. However, future studies on teacher personality should consider examining both the immediate and the long-term effects of a teacher’s personality on students’ education and job-related outcomes.

The field of teacher personality is expanding, and the potential implications of such research are exciting. Our study aimed to summarize the current research that has examined teacher personality in relation to its effect on teacher effectiveness and burnout. Much more research is necessary to understand its place in teaching and learning before its appropriate use can be determined. Future academic endeavors in the area of teacher personality would not only be relevant and beneficial to students, but also to teachers and the education system as a whole.
